# Species Level Description of the Human Ileal Bacterial Microbiota

**DOI:** 10.1038/s41598-018-23198-5

**Published:** 2018-03-16

**Authors:** Heidi Cecilie Villmones, Erik Skaaheim Haug, Elling Ulvestad, Nils Grude, Tore Stenstad, Adrian Halland, Øyvind Kommedal

**Affiliations:** 1 Department of Microbiology, Vestfold Hospital Trust, 3103 Tønsberg, Norway; 2 Department of Urology, Vestfold Hospital Trust, 3103 Tønsberg, Norway; 3 Department of Microbiology, Haukeland University Hospital, 5021 Bergen, Norway; 4 Department of Clinical Science, University of Bergen, 5021 Bergen, Norway; 5 Department of Infectious Medicine, Vestfold Hospital Trust, 3103 Tønsberg, Norway

## Abstract

The small bowel is responsible for most of the body’s nutritional uptake and for the development of intestinal and systemic tolerance towards microbes. Nevertheless, the human small bowel microbiota has remained poorly characterized, mainly owing to sampling difficulties. Sample collection directly from the distal ileum was performed during radical cystectomy with urinary diversion. Material from the ileal mucosa were analysed using massive parallel sequencing of the 16S rRNA gene. Samples from 27 Caucasian patients were included. In total 280 unique Operational Taxonomic Units were identified, whereof 229 could be assigned to a species or a species group. The most frequently detected bacteria belonged to the genera *Streptococcus*, *Granulicatella*, *Actinomyces*, *Solobacterium*, *Rothia*, *Gemella* and *TM7(G-1)*. Among these, the most abundant species were typically streptococci within the *mitis* and *sanguinis* groups, *Streptococcus salivarius*, *Rothia mucilaginosa* and Actinomyces from the *A*. *meyeri/odontolyticus group*. The amounts of Proteobacteria and strict anaerobes were low. The microbiota of the distal part of the human ileum is oral-like and strikingly different from the colonic microbiota. Although our patient population is elderly and hospitalized with a high prevalence of chronic conditions, our results provide new and valuable insights into a lesser explored part of the human intestinal ecosystem.

## Introduction

The human gut microbiota has been extensively investigated in recent years owing to its impacts on human health and disease^1–3^. Most research on host-microbe interactions are based on studies of the faecal microbiota. The feasibility of this practice is questionable^4–6^. The small bowel is responsible for most of the body’s nutritional uptake and for the development of intestinal and systemic tolerance towards microbes. It has protruding villi, making its surface area approximately fifteen times greater than that of the colon^7^. The small intestines also have a thinner, more vulnerable mucin barrier than the colonic epithelium, offering closer contact between the luminal content and mucosal enteroendocrine and immunological cells^8,9^. Peyer’s patches are present mainly in the distal jejunum and ileum and increase to a maximum together with the Paneth cells in the distal ileum^10–12^.

A recent study on rats have concluded that faecal sampling misrepresents the microbiota at different intestinal locations^13^. Similar studies have been difficult to perform in humans, due to major challenges in the procurement of representative and uncontaminated samples from intestinally healthy individuals. Sampling techniques have included naso-ileal catheters^14^, capsules^15,16^ and retrograde colonoscopy^16,17^. Investigations have also been performed on ileostomy effluent samples^12,14,18,19^, autopsies^20^ and samples from patients suffering from inflammatory bowel disease or in need of emergency surgery^21–23^.

Patients undergoing radical cystectomy, a treatment where the bladder is removed, have their urinary diversion created using a segment of ileum. Most of these patients have no known intestinal diseases, despite their high median age and bladder condition. In this study, by using uncontaminated samples collected directly from the distal ileal mucosal surface during this procedure, we aimed to characterize the microbiota of the ileum using massive parallel sequencing of the bacterial gene encoding 16S ribosomal RNA (16S rRNA gene). When the 16S rRNA gene provided insufficient species-level discrimination, we supplemented the characterization with targeted sequencing of alternative genes providing higher taxonomic resolution. Species-level identification is necessary to increase our understanding of the microbial ecosystem and host-microbe interactions, but also the microbiota’s role in infections through leaking, translocation and haematogenous spread of potentially pathogenic organisms.

## Results

The mean and median age of the 27 patients was 71 years (range 54–86). Indications for cystectomy were bladder cancer for 24 patients, complications following prostate cancer for two patients and chronic cystitis for one. Nine patients received neoadjuvant chemotherapy prior to surgery but none had received radiotherapy or immunotherapy. Most suffered from additional chronic diseases and 22 out of 27 used medications on a regular basis. Fifteen out of 27 had at least one antibiotic treatment during the last six months prior to admission, mainly for urinary tract infections. Further demographic and clinical data are displayed in Table 1.

**Table 1:** Demographic and clinical data.

Population characteristics	Years/ kg/m2	(range)	Patient identification, sample number
**Age**, **median years (min-max)**	71	(53–85)	
**BMI**, **median kg/m**^**2**^ **(min-max)**	27	(21–40)	
**BMI ≥ 30**	5		4, 5, 10, 21, 29
**BMI ≥ 40**	1		29
**Population characteristics**	**Number of patients**	**(%)**	**Patient identification**, **sample number**
**Sex**, **male**	21	(78)	
**Sex**, **female**	6	(22)	9, 13, 14, 15, 16, 20
**Neoadjuvant chemotherapy**	8	(29)	
MVAC	5	(19)	3, 9, 14, 20, 28
GC	3	(11)	10,11,16
**Antibiotic prophylaxis**	27	(100)	
quinolone + metronidazole	25	(93)	
trimethoprim sulfa + metronidazole	1	(4)	13
furadantin + metronidazole	1	(4)	18
**Indication**, **bladder cancer**	24	(88)	
**Indication**, **prostate cancer**	2	(7)	2, 27
**Indication**, **chronic cystitis**	1	(4)	15
**Chronic diseases none**	5	(19)	8, 11, 14, 18, 30
**Chronic diseases yes**	22	(81)	
Diabetes mellitus 2	2	(7)	10, 22
Cardiovascular disease*	15	(56)	1, 2, 3, 4, 5, 9, 10, 13, 16, 19, 20, 22, 23, 24, 28
COPD/Asthma	5	(19)	5, 6, 12, 28, 29
Irritable bowel disease	1	(4)	20
Constipation	1	(4)	28
Cancer coli operata	1	(4)	6
**Regular medications**, **none**	5	(19)	8, 11, 14, 18, 30
**Regular medications**, **yes**	22	(81)	
Statins	15	(56)	1, 2, 4, 5, 10, 13, 16, 19, 21, 22, 24, 26, 27,28, 29
PPI	3	(11)	3, 26, 27
Antidiabetics	2	(7)	10, 22
**Antibiotics prior to admission (last week)**	5	(19)	11, 13, 15, 16, 19
**Antibiotics prior to admission (last six months)**	15	(56)	1, 2, 9, 11, 12, 13, 14, 16, 18, 19, 20, 26, 28, 29, 30

BMI: body mass index, MVAC: Methotrexate, vinblastine, neomycin (Adriamycin), cisplatin. GC: Gemcitabine, cisplatin. COPD: Chronic Obstruction Pulmonary Disease, *including hypercholesterolemia and hypertension

The mean number of reads was 318,742 per sample (Supplementary Table S1). After removal of short reads (<250 base pairs), small clusters (<10 reads) and chimeras, a mean of 100,276 valid reads remained (range 15,079 to 376,370, median 97,047) for each sample. The main reason for this substantial loss was short reads, probably due to premature truncation in the ion semiconductor sequencing technology^24^. About 50% of the reads in all samples had a length below 250 base pairs.

**Figure 1:**
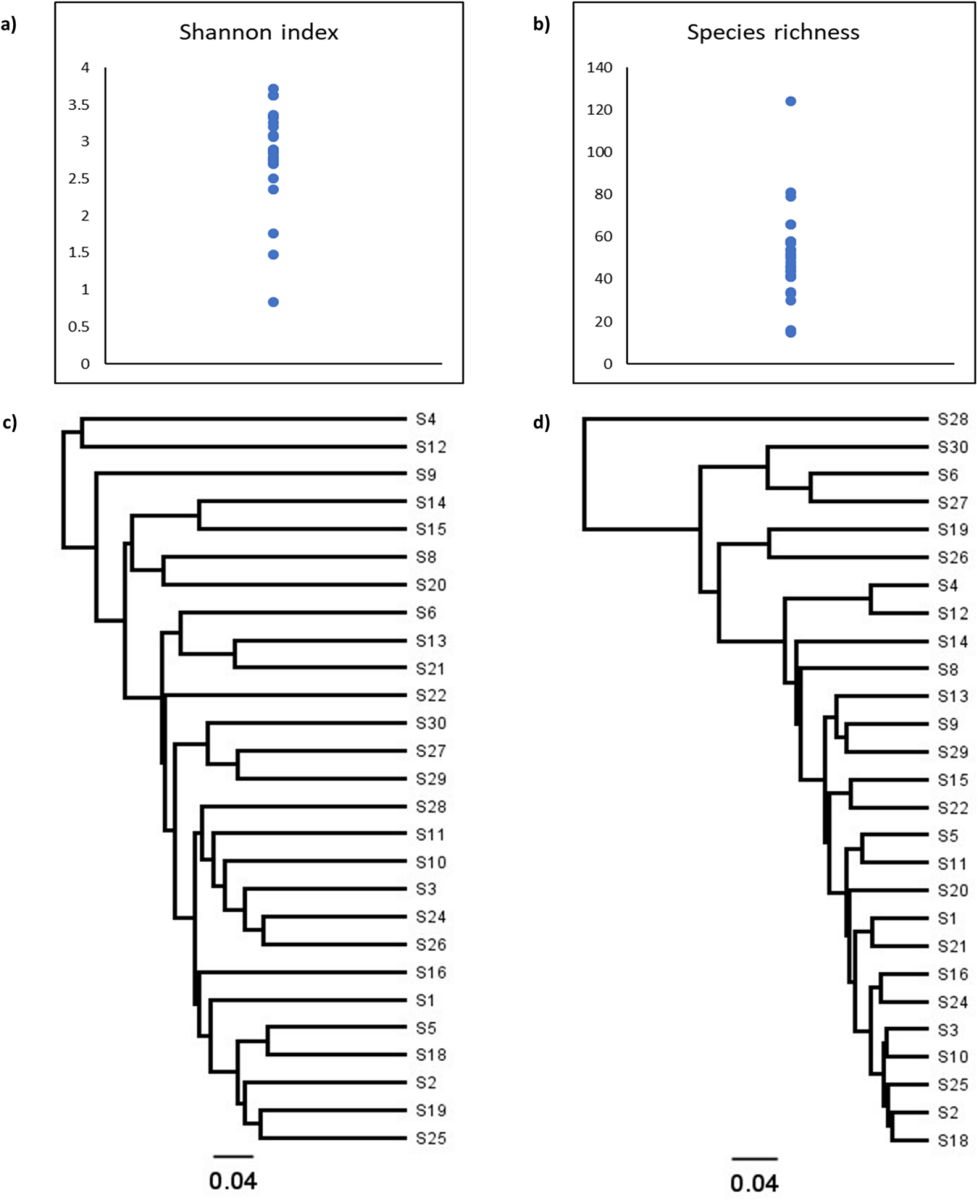
Alpha- and beta diversities. (**a**) Shannon index distribution. (**b**) Species richness distribution. (**c**) Unweighted (qualitative) UniFrac analyses. (**d**) Weighted (quantitative) UniFrac analyses.

By 16S rRNA sequencing, a total of 280 unique Operational Taxonomic Units (OTU’s) were accepted, whereof 191 could be identified to the species level, 38 to a species group level, and 51 to the genus level (Supplementary Table S2). The detailed results from the targeted Sanger-sequencing of gdh and rpoB-genes, providing species level identification for some of the bacteria that could only be assigned to the group level by 16S, are provided in Supplementary Table S3. Whenever species level identifications obtained by these supplementary genes are used in the text, the gene is indicated in parenthesis after the species name. The mean species richness was 51 (range 15 to 124, median 50) with an average Shannon index of 2.84 (range 0.83 to 3.72, median 2.56). Most samples had species richness between 30 and 79 and a Shannon-index between 2.36 and 3.72 (Fig. 1a,b and Supplementary Table S4).

### Bacterial structure of the ileal samples

**Figure 2:**
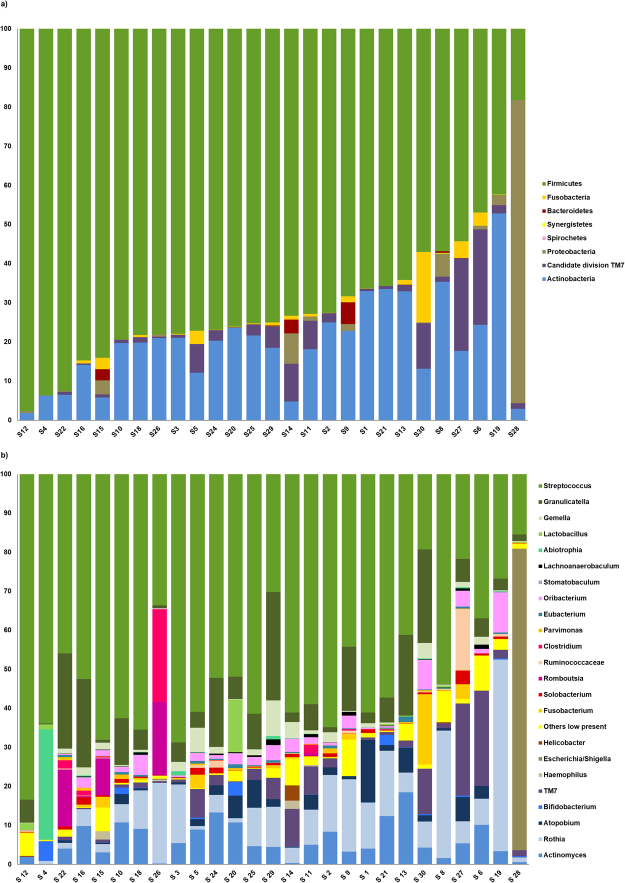
Phylum and genus distribution. (**a**) Phylum distribution. Relative abundances based on number of reads (%). (**b**) Distribution of most abundant genera. Relative abundances based on number of reads (%).

The most abundant phylum in the distal ileum was Firmicutes followed by Actinobacteria. Most samples also contained Candidate division TM7 (24/27), Proteobacteria (24/27) and Fusobacteria (23/27) (Fig. 2a). In four samples species from the latter three phyla flourished at the expense of both Firmicutes and Actinobacteria. These were *Fusobacterium periodonticum* in sample 30, *TM7(G-1) sp*. *oral taxon 352* in samples 6 and 27 and *Escherichia coli* in sample 28. Although outliers in the weighted UniFrac analysis, they remained within the main cluster in the unweighted UniFrac analysis (Fig. 1c,d), reflecting that the qualitative species compositions were not atypical. The most extreme outlier, sample 28 with 77% reads from *E*. *coli*, was from a patient with long term constipation that had been using a combination of high-osmotic and contact-laxative medications on a regular basis. We observed no congruence between clusters in the UniFrac analyses and BMI category, gender, use of statins, antibiotic treatment or neoadjuvant chemotherapy. Bacteroidetes were found in 11 out of 27 samples whereas species within the phyla Syngergistetes, Tenericutes and Spirochetes were detected at low levels in only one sample each.

**Figure 3:**
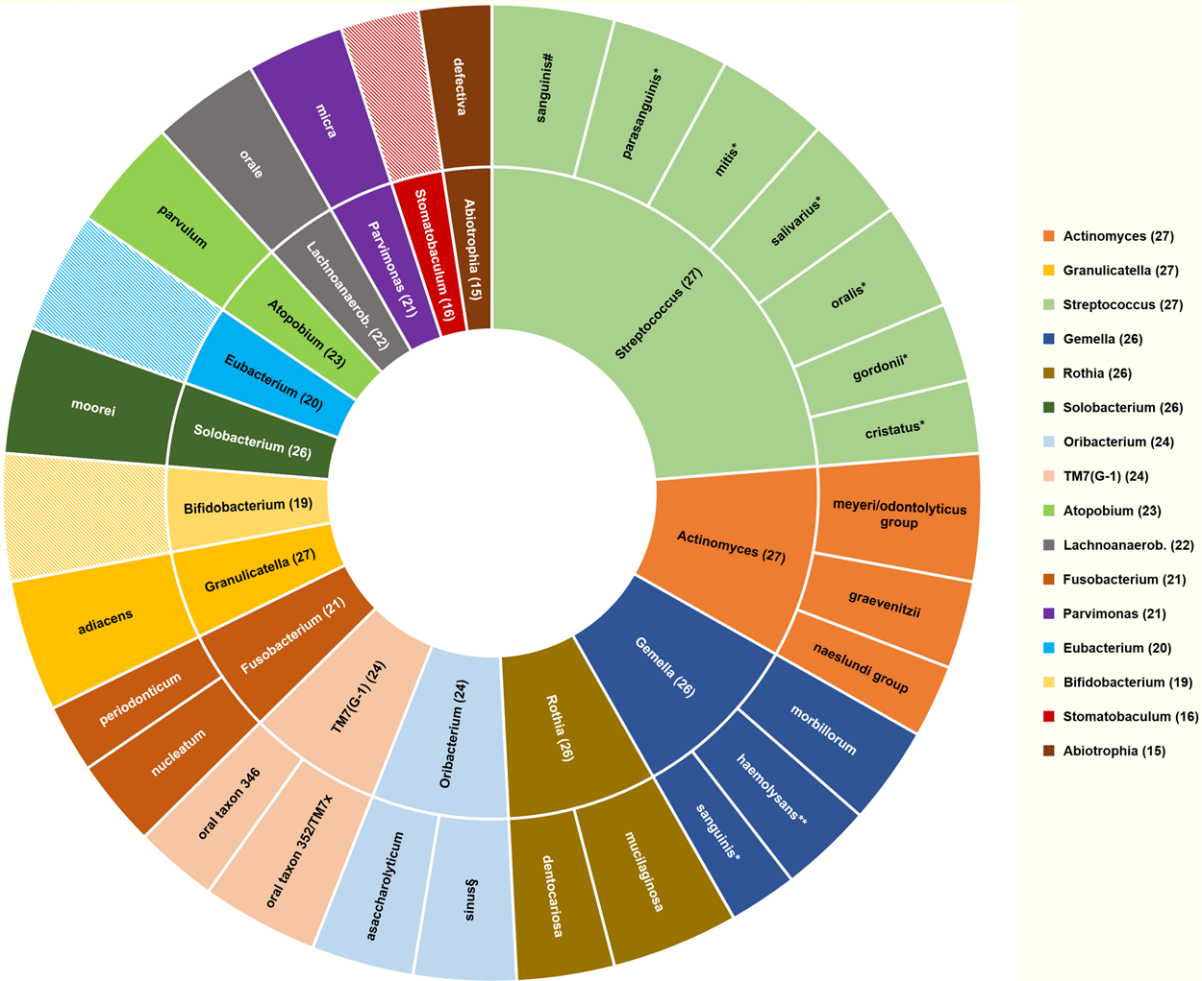
Core microbiota of the ileum. The inner circle represents the genus-level core microbiome defined as genera present in >50% of samples. The outer circle represents the species-level core microbiome defined as species present in >50% of samples. For the outer circle, the width of a segment is proportional to the observed incidence for that species. *Species level identification obtained with targeted gdh or rpoB Sanger sequencing. **Species level identification obtained with rpoB sequencing. Includes *Gemella haemolysans* sensu strictu (n = 7) and the newly proposed species *Gemella para-haemolysans* (n = 9) and Gemella taiwanensis (n = 6). ^#^Only 0.7% distance to *Streptococcus sinensis*. Formally *S*. *sanguinis (S*. *sinensis)*. ^§^Only 0.7% distance to *Oribacterium parvum*. Formally *O*. *sinus (O*. *parvum)*.

The distal ileum core microbiota at genus and species level, defined as genera and species that occurred in more than 50% of the individuals, are displayed in Fig. 3. The typical microbiota is dominated by the facultative anaerobic genera *Streptococcus*, *Actinomyces*, *Gemella*, *Granulicatella* and *Rothia* and in most cases also contain the genera *Atopobium*, *Lachnoanaerobaculum*, *Oribacterium*, *Solobacterium* and *TM7(G-1)* (Fig. 2b). On average, strict anaerobic bacteria constituted only 10% of the reads (range 0.5% to 29%, median 7%), whereof the most significant were *Atopobium parvulum*, *Oribacterium asaccharolyticum*, *Oribacterium sinus*, *Solobacterium moorei*, *Fusobacterium nucleatum*, *Fusobacterium periodonticum*, *Parvimonas micra* and *Bifidobacterium longum*. *Clostridium* species were detected only sporadically and at low levels, except from *Clostridium celatum*, which was found in seven samples. *Clostridium celatum* was also the dominant anaerobe in one individual. The newly described species *Romboutsia timonensis* (Clostridiales order), was recovered from six samples and among the dominant anaerobes in three. *Prevotella* and *Bacteroides* species were found in only six and four samples respectively. *Bacteroides fragilis* was only detected in one patient with 0.01% of the total reads. *Faecalibacterium prausnitzii*, considered a dominant and ubiquitous member of the human intestinal flora^25^, was detected in only two patients with <0.1% of the total reads. Microaerophilic bacteria were represented by *Helicobacter pylori* and *Campylobacter* species, mainly *Campylobacter concisus*. Strict aerobic bacteria were present in small fractions in seven samples except for sample 19 with 2.0% of reads representing *Stenotrophomonas maltophila*.

## Discussion

In this study we have identified a group of patients that provides surgical access to the ileal lumen and that potentially represents an attractive population for future research on host-microbiota interactions. To obtain species level identification we chose a bioinformatic approach specifically developed for use in diagnostic microbiology.

The study reveals fundamental differences between the microbiota of the distal ileum and the colon. This contradicts previous studies based on samples collected by retrograde colonoscopy which indicate that the small bowel microbiota at the level of the distal ileum is similar to the colonic microbiota^16,17^. Our findings also diverge significantly from the other small and inconsistent studies on samples from ileostomy patients or collected from healthy individuals using naso-ileal catheters^12,14,15,18,19^. The colonic content is dominated by the Firmicutes and Bacteroidetes phyla^4,26–28^. Although Firmicutes is the major phylum also in ileum, it is here represented mainly by facultative anaerobic species within the Bacilli class (Streptococcaceae, Lactobacillacae, Aerococcaceae, Carnobacteriaceae) and not the strict anaerobic Firmicutes from the Clostridia class (Peptostreptococcaceae, Eubacteriaceae, Clostridiaceae, Lachnospiraceae, Ruminococcacea) found in colon (Fig. 2b). In our samples, Clostridia account for less than 5% of the sequences whenever present. The second most dominant phylum in ileum was Actinobacteria whereas species from the Bacteroidetes phylum were detected only sporadically and at low levels. The microbiota of the distal ileum has a higher resemblance to the oral microbiota with dominance of viridans streptococci and high contributions of *Rothia*, *Gemella*, *Granulicatel metagenomic sequencing was performed at the la* and *Actinomyces* species. The major differences are higher abundances of *Neisseria*, *Haemophilus*, *Prevotella* and *Veillonella* in the oral cavity and higher levels of the TM7 phylum in the ileum^28–32^. A study using 16S metagenomics to analyse capsule biopsies from jejunum^15^ also found similarity with the oral microbiota. In jejunum even the contributions of *Haemophilus*, *Prevotella* and *Veillonella* were preserved. The same study demonstrated an inverse relationship between *Streptococcus* and *Prevotella* abundances, supporting the almost complete absence of *Prevotella* in our *Streptococcus*-dominated ileal specimens.

Twenty-four individuals harboured TM7 genera (3–11 species each), representing almost a quarter of the reads in some samples. Although previously detected in the oral cavity and colon, the TM7-phylum has not previously been reported from the small intestine. The most likely explanation for this discrepancy is methodological. Microarrays designed for the gut-microbiome like the HITChip^33^ or HuGChip^34^ do not target the TM7 phylum. In sequence based studies, the lack of TM7-references in databases might have rendered TM7-derived reads unassigned or even erroneously assigned to other phyla. Only two reports exist on the successful cultivation of TM7-species from human samples. In the first study^35^, several TM7-species were identified in mixed bacterial cultures together with *Actinomyces naeslundi* group, *Fusobacterium nucleatum*, *Parvimonas micra*, *Shuttleworthia satelles*, *Streptococcus gordonii* and *Veillonella parvula*, all frequently detected in this study as well. Dual-species biofilm experiments demonstrated synergistic biofilm formation with *F*. *nucleatum*, *P*. *micra* and *S*. *gordonii*.(gdh) Interestingly, the cellular shape of the TM7-species shifted between coccoid and filamentous depending on which bacterium it was co-cultivated with. In the second study^36^, a TM7-species was recovered as an epibiont of a specific clone of *Actinomyces odontolyticus*. Together, this creates the impression of a phylum with potential for close interactions with its neighbours. Establishing the human ileum as a major TM7-reservoir represents an intriguing novel discovery that should stimulate research on this enigmatic phylum.

An interesting finding was the presence of *Helicobacter pylori* in three ileal samples, representing as much as 3.9% of the 16S sequences in sample 14. It has until now been thought to colonize only the gastric mucosa. Additional studies, including fluorescence *in situ* hybridization of biopsies, need to be undertaken to further illuminate *H*. *pylori* colonization of the ileum.

Colonic and ileal microbiota are reported to change with diet^14,37,38^. A strength of our study is therefore that the collection of mucosa-near samples under standardized preoperative diet restrictions assured that microbiotas were compared under similar nutritional conditions. However, we only obtain a snapshot of the ileal microbiota under these specific conditions. Theoretically, other species could dominate after the intake of more protein-rich or fat-rich food.

Despite the almost complete absence of known intestinal illnesses in our patient population, the samples obtained are not necessarily representative of a normal ileal flora. The median age was high and most patients suffered from chronic conditions including COPD and cardiovascular diseases. These conditions themselves as well as some of the medications prescribed to treat them could affect the intestinal microbiota. Another concern is the impact of the preoperative antibiotic prophylaxis. Antibiotics are documented to impact gut microbiota^39,40^. It can be argued that the luminal surface of the ileum is covered with a mucus layer^21^ likely to protect bacteria against both direct and systemic effects of antibiotics at least in the short-term. It can also be argued that results from DNA-based analyses are less vulnerable to short-term effects of antibiotics due to detection of DNA from non-viable bacteria and even persisting free DNA from lysed bacterial cells. However, the exact impact of the antimicrobial exposure in our population remains unknown. In the UniFrac analysis, patients that had received antibiotics during the last six months or during the last week prior to surgery did not cluster separately from the remaining population.

Patients undergoing cystectomy are currently the closest we might get to a “normal” population. Obtaining clean surgical samples for unbiased metagenomic characterization from this group, presents no major ethical dilemmas. However, high median age, underlying cancer, chronic illnesses and antibiotic prophylaxis could all impact microbiota composition. Some of the differences observed between this and previous studies could arise from these factors.

In conclusion, the distal part of ileum harbours a distinctive niche of the human gut ecosystem that differs more from the colonic than the salivary flora. These findings oppose the relevance of the bacterial flora in colon as proxy for the overall intestinal microbiota. In future studies of host-microbe interactions, it will be necessary to pay greater attention to the ileal microbiota.

## Materials and Methods

### Population and sample collection

Patients undergoing cystectomy with urinary diversion in Vestfold Hospital Trust (Tønsberg, Norway) are enrolled in a local quality registry based on broad informed consent. Informed consent was obtained from all subjects. Thirty patients were consecutively included in the current survey that was approved by the Regional Ethical Committee of Southern and Eastern Norway Regional Health Authority (2016/926 REK Sør-Øst D) and performed in accordance with European Association of Urology (EAU) and local guidelines and regulations.

All included patients were Caucasian. None of the patients had current gastrointestinal diseases except from patient number 20 with irritable bowel syndrome and patient number 28 with chronic obstipation (Table 1). Due to low output of reads after sequencing, three of the patients (number 7, 17 and 23) were later excluded (Supplementary Table S1). Patients were routinely fasting for solid food for 20 hours prior to surgery. They were given standardized carbohydrate drinks (PreOp Nutricia, Danone, The Netherlands) the evening before and the morning prior to surgery, and otherwise followed the Enhanced Recovery After Surgery (ERAS)-regimen^41^. Two patients with diabetes (patient 10 and 22) did not receive PreOp drinks. All patients received peroral ofloxacin 400 mg (25 patients) prophylaxis two hours before surgery or according to findings in preoperative urine-culture (Table 1). Metronidazole 1000 mg along with tranexamic acid 1000 mg was administered intravenously from the start of surgery as standard.

The surgeon collected the sample about 25 cm proximal to the ileocecal valve by rubbing the swab against the luminal wall, and then inserted the swab in a standardized Transwab medium (MWE, Medical Wire, England). Samples were frozen immediately at minus 70 °C for later DNA extraction.

### Pre-PCR treatment of samples

The collected sample-suspension was diluted 1:2 with Qiagen ATL buffer (Qiagen, Hilden, Germany) and extracted using the “Pathogen complex kit” (Qiagen) according to the manufacturer’s instruction on a QIAsymphony platform (Qiagen). Negative controls from the relevant batches of Transwab media were extracted in the same manner.

### 16S metagenomic analysis

16S metagenomic sequencing was performed at the Public Health Agency of Sweden that offers this as a commercial service for the Nordic countries. Briefly, a fragment containing the variable areas V3 and V4 was amplified using forward-primer 5′-CGG-CCC-AGA-CTC-CTA-CGG-GAG-GCA-GCA-3′ and reverse-primer 5′-GCG-TGG-ACT-ACC-AGG-GTA-TCT-AAT-CC-3′^42^. An Ion Chef instrument (Thermofisher, Foster City, California) was used for automated library preparation and bidirectional sequencing was done using the IonS5 sequencer (Thermofisher). Barcode separated FASTQ-files were processed individually using the RipSeq NGS software^43^ (Pathogenomix, Santa Cruz, California). Reads shorter than 250 base pairs were removed before de-novo clustering into OTU’s using a similarity threshold of 99%. OTUs containing less than 10 sequences were rejected. For each of the remaining OTUs the most representative sequence variant was used for a BLAST-search against the curated “RipSeq. 16S human pathogen”-database (Pathogenomix) that currently contains more than 2500 non-redundant references for clinically relevant bacteria and commensals. OTU’s that did not obtain a species-level match were re-analysed against GenBank (nr/nt-database) and the “Human oral microbiome” database (www.homd.org) assuring the highest possible level of identification for all clusters. RipSeq NGS flags the quality of a BLAST-result based on adjustable interpretation criteria. For unambiguous species-level identification, the Clinical and Laboratory Standard Institute (CLSI) guidelines for 16S sequence interpretation recommends ≥99% homology with a high-quality reference combined with a minimum distance of >0.8% to the next alternative species^44^. These recommendations are based on the V1-V3 segment. Due to the lower overall variability in the V3-V4 segment we adopted a more stringent cut off of ≥99.3% while retaining the minimum distance to the next species at >0.8%. OTU’s with a similarity >99,3% but with a distance to next species of ≤0,8% was assigned to a group of species (group-level identification) whereas OTU’s with a similarity of 97–99.2% was assigned to the genus level. When appropriate, the provisional taxonomy developed by the Human oral microbiome project (www.homd.org) was used for hitherto unnamed species^45^. Chimeras and any species/OTU found in the negative controls were rejected. A comparison between results obtained by QIIME, the software package typically used in 16S rRNA metagenomic studies^46^ and RipSeq NGS on a commercial Mock-up community is provided in supplementary table S5. This comparison demonstrates the better performance of the RipSeq NGS software in obtaining species level identification.

Comparative analyses were performed using the QIIME. Alfa-diversities were measured using the Shannon-index and weighted and unweighted UniFrac analyses were used to calculate beta-diversities^47,48^.

### Sanger sequencing of alternative genes

For discrimination among bacteria with too similar 16S-amplicons, we designed species-specific PCRs for genes with higher mutations rates (Supplementary Table S6). Due to limited volumes of DNA-eluate we prioritized species that consistently came out among the top-scoring organisms in frequently encountered ambiguous 16S clusters. For streptococci, the gene for glutamate dehydrogenase (gdh) was selected and we applied the taxonomical modifications proposed by Jensen *et al*.^49^. Within the mitis group, we targeted *Streptococcus oralis* (including ssp. *dentisani*, *oralis* and *tigurinus*) and *Streptococcus mitis*. For *Streptococcus infantis* a single gdh-PCR could not be designed due to varying sequence patterns. Recent whole genome-based comparisons of strains within the *S*. *infantis* clade expose significant unresolved taxonomical matters^49^. Within the parasanguinis group, PCR’s were designed for *Streptococcus cristatus* (synonym: *S*. *oligofermentans*), *S*. *parasanguinis* and *S*. *gordonii*. *Streptococcus salivarius* and *S*. *vestibularis* were targeted by a shared PCR and discriminated by the nucleotide sequence. *S*. *sanguinis* was unambiguously identified based on the 16S rRNA-gene.

For discrimination within the *Gemella haemolysans/sanguinis* cluster the gene for RNA-dependent polymerase beta (rpoB) was used. In addition to specific PCRs for *G*. *haemolysans* and *G*. *sanguinis* we designed a shared PCR for *G*. *haemolysans* and the newly proposed species *Gemella parahaemolysans* and *Gemella taiwanensis*^50^.

All primers were aligned against available references in GenBank (nr/nt and wgs databases) to assure coverage of all known sequence variants. The PCR-products from positive reactions were sequenced using Sanger-sequencing and aligned against the GenBank nr/nt and wgs databases for confirmation. Due to lack of established cut-offs for a valid species level identification all species level assignments were supported by a pairwise comparison of the alignment table in GenBank using the “distance tree of results” function.

Primer sequences, primer concentrations and PCR conditions are listed in Supplementary Table S6. SYBR-green real-time PCRs were performed in 25 µl reaction tubes on a SmartCycler real-time apparatus (Cepheid, Sunnyvale, California). PCR mixtures consisted of 12.5 µl ExTaq SYBR master mix (TaKaRa, Otsu, Japan), 0.4 or 0.6 µM of each primer (corresponding to 1.0 or 1.5 µl from a 10 µl stock solution), 8.0 or 8.5 µl PCR-grade water (depending on primer-volume) and 2 µl extracted DNA. Thermal profiles included an initial polymerase activation step at 95 °C for 30 seconds, followed by 45 cycles of 95 °C for 10 seconds, PCR-specific annealing temperature for 10 seconds and 72 °C for 20 seconds.

The products from positive PCR reactions were spun out of the SmartCycler reaction tubes into a 1.5-ml Eppendorf tube and cleaned up using ExoSAP-IT enzymatic degradation (Affymetrix, Santa Clara, California). Cycle sequencing was run for 28 cycles with annealing temperature 50 °C for all amplicons. Sanger sequencing was performed using an ABI prism 1.1 Big Dye sequencing kit and an ABI 3730 DNA analyzer (Applied Biosystems, Foster City, California 16S).

### Data availability

Source data of this study are available from the corresponding author upon reasonable request. Not all patient data are publicly available due to restrictions from the Regional Ethical Committee (REK Sør-Øst D).

## Electronic supplementary material

Supplementary information
