# Supplementary material for: Species Level Description of the Human Ileal Bacterial Microbiota
Source: Sci Rep. 2018 Mar 16;8:4736. doi: 10.1038/s41598-018-23198-5 (PMC5856834; doi:10.1038/s41598-018-23198-5)
Supplement: Supplementary file 1 — Supplementary information [file 41598_2018_23198_MOESM1_ESM.docx]

**SUPPLEMENTARY INFORMATION**

**Title:**

**SPECIES LEVEL DESCRIPTION OF THE HUMAN ILEAL BACTERIAL MICROBIOTA**

**AUTHOR INFORMATION**

Villmones, Heidi Cecilie^1^; Haug, Erik Skaaheim^2^; Ulvestad, Elling^3,4^; Grude, Nils^1^; Stenstad, Tore^5^, Halland, Adrian^2^ and Kommedal, Øyvind^3^

1. Department of Microbiology, Vestfold Hospital Trust, 3103 Tønsberg, Norway.
2. Department of Urology, Vestfold Hospital Trust, 3103 Tønsberg, Norway.
3. Department of Microbiology, Haukeland University Hospital, 5021 Bergen Norway.
4. Department of Clinical Science, University of Bergen, 5021 Bergen, Norway.
5. Department of Infectious Medicine, Vestfold Hospital Trust, 3103 Tønsberg, Norway.

**Corresponding author:**

Heidi Cecilie Villmones, Department of Microbiology,

Vestfold Hospital Trust, Postbox 2168, 3103 Tønsberg, Norway

E-mail: [heivil@siv.no](mailto:heivil@siv.no)

Telephone number 0047 92214631 or 0047 33346598

**Supplementary Table S1. CT-values from the universal 16S rRNA-PCR and technical data from the metagenomic analysis**

| Sample/patient number | CT 16S | Cluster 10c1p | Used reads | Total reads |
| --- | --- | --- | --- | --- |
| 1 | 24,2 | 183 | 157,399 | 447,090 |
| 2 | 23 | 159 | 81,872 | 242,510 |
| 3 | 21,4 | 362 | 376,370 | 1,060,039 |
| 4 | 25,1 | 59 | 45,722 | 127,220 |
| 5 | 21,7 | 135 | 59,851 | 181,708 |
| 6 | 31 | 61 | 15,079 | 55,545 |
| **7** | **31** | **22** | **2,233** | **10,692** |
| 8 | 28,7 | 131 | 99,875 | 279,138 |
| 9 | 26 | 126 | 72,423 | 230,495 |
| 10 | 17,5 | 196 | 119,260 | 380,629 |
| 11 | 25,7 | 126 | 97,047 | 335,058 |
| 12 | 25,7 | 122 | 14,938 | 79,404 |
| 13 | 27,7 | 118 | 53,053 | 194,219 |
| 14 | 25 | 138 | 58,427 | 224,234 |
| 15 | 19,4 | 284 | 185,680 | 705,976 |
| 16 | 24,4 | 59 | 59,991 | 187,406 |
| **17** | **31,1** | **19** | **2,532** | **10,750** |
| 18 | 19,8 | 136 | 59,929 | 228,237 |
| 19 | 27,2 | 65 | 46,223 | 149,186 |
| 20 | 24,2 | 137 | 99,054 | 296,087 |
| 21 | 24,6 | 107 | 101,140 | 278,633 |
| 22 | 24,5 | 221 | 152,484 | 498,152 |
| **23** | **30,4** | **83** | **24,256** | **80,232** |
| 24 | 21,1 | 153 | 102,794 | 338,886 |
| 25 | 21,1 | 105 | 89,416 | 283,769 |
| 26 | 18,1 | 256 | 129,408 | 463,484 |
| 27 | 21,5 | 153 | 103,337 | 376,853 |
| 28 | 24,1 | 103 | 102,493 | 259,576 |
| 29 | 24,9 | 112 | 67,555 | 23,1127 |
| 30 | 24,9 | 201 | 156,623 | 47,1374 |
| Neg ctr 0B | 32,3 | 11 | 4,958 | 16,279 |
| Neg ctr 0A | 33,1 | 20 | 3,661 | 12,709 |
| FOHMnegCtr |  | 14 | 3,964 | 15,293 |

CT 16S: Cycle Threshold in the universal 16S PCR; Cluster 10c1p: Number of clusters with above 10 reads and less than one percent variation; Used reads: The final number of reads after removal of short reads, small clusters and chimeras; Total reads: The total sequence output for each sample

**Supplementary Table 2: Alphabetical list of OTU´s and species obtained by 16S sequencing**

| OTUs / SPECIES | Samples of total 27 | Mean | Median | Min | Max |
| --- | --- | --- | --- | --- | --- |
| *Abiotrophia defectiva* | 15 | 1,18 | 0,17 | 0,01 | 28,15 |
| *Abiotrophia (genus only)* | 2 | 0,01 | 0,09 | 0,03 | 0,15 |
| *Achromobacter (genus only)* | 3 | 0,02 | 0,03 | 0,02 | 0,41 |
| *Acinetobacter baumannii* | 1 | NA | NA | 0,05 | 0,05 |
| *Actinobaculum sp. oral taxon 183* | 2 | NA | NA | 0,01 | 0,08 |
| *Actinomyces cardiffensis* | 2 | NA | NA | 0,01 | 0,01 |
| *Actinomyces dentalis* | 1 | NA | NA | 0,01 | 0,01 |
| *Actinomyces (genus only)* | 17 | 0,29 | 0,16 | 0,01 | 1,82 |
| *Actinomyces gerencseriae* | 3 | 0 | 0,01 | 0,01 | 0,02 |
| *Actinomyces graevenitzii* | 18 | 0,43 | 0,06 | 0 | 3,79 |
| *Actinomyces naeslundi group* | 15 | 0.66 | 0,11 | 0,02 | 15,69 |
| *Actinomyces meyeri/odontolyticus group* | 26 | 4,32 | 3,67 | 0,03 | 12,84 |
| *Actinomyces oral taxon 178* | 9 | 0,06 | 0,02 | 0,01 | 1,31 |
| *Actinomyces oral taxon 448* | 8 | 0,04 | 0,04 | 0,01 | 0,5 |
| *Actinomyces oral taxon 848* | 2 | NA | NA | 0,01 | 0,03 |
| *Actinomyces massiliensis* | 3 | 0 | 0,01 | 0,01 | 0,04 |
| *Actinomyces sp. (sp4-iso1_H03x4)* | 10 | 0,26 | 0,41 | 0,02 | 2,79 |
| *Actinomyces turicensis* | 1 | NA | NA | 0,01 | 0,01 |
| *Aerococcus christensenii* | 1 | NA | NA | 0,03 | 0,03 |
| *Afipia (genus only)* | 2 | NA | NA | 0,07 | 0,15 |
| *Aggregatibacter segnis* | 2 | NA | NA | 0,01 | 0,04 |
| *Aggregatibacter aphrophilus* | 1 | NA | NA | 0,03 | 0,03 |
| *Aggregatibacter (genus only)* | 1 | NA | NA | 0,04 | 0,04 |
| *Aggregatibacter oral taxon 458* | 2 | NA | NA | 0,02 | 0,1 |
| *Aggregatibacter oral taxon 513* | 1 | NA | NA | 0,06 | 0,06 |
| *Alistipes finegoldii* | 2 | NA | NA | 0,13 | 0,52 |
| *Alistipes putredinis* | 1 | NA | NA | 0,69 | 0,69 |
| *Alloprevotella tannerae* | 1 | NA | NA | 0,02 | 0,02 |
| *Alloprevotella oral taxon 308* | 1 | NA | NA | 0,11 | 0,11 |
| *Alloprevotella oral taxon 473* | 1 | NA | NA | 0,01 | 0,01 |
| *Alloscardovia omnicolens* | 12 | 0,15 | 0,16 | 0,01 | 1,58 |
| *Atopobium deltae (A. minutum)* | 2 | NA | NA | 0,01 | 0,08 |
| *Atopobium (genus only)* | 12 | 0,04 | 0,02 | 0 | 0,39 |
| *Atopobium parvulum* | 23 | 2,35 | 1,53 | 0,04 | 15,88 |
| *Atopobium rimae* | 12 | 0,05 | 0,02 | 0,01 | 1,12 |
| *Atopobium vaginae* | 2 | NA | NA | 0,03 | 0,04 |
| *Bacterioidales(G-2) oral taxon 274* | 2 | NA | NA | 0,01 | 0,05 |
| *Bacteroides caccae* | 2 | NA | NA | 0,01 | 0,45 |
| *Bacteroides dorei (B. vulgatus)* | 2 | NA | NA | 0,03 | 1,78 |
| *Bacteroides faecis* | 1 | NA | NA | 0,33 | 0,33 |
| *Bacteroides fragilis* | 1 | NA | NA | 0,01 | 0,01 |
| *Bacteroides massiliensis* | 1 | NA | NA | 0,76 | 0,76 |
| *Bacteroides stercoris* | 1 | NA | NA | 0,5 | 0,5 |
| *Bacteroides uniformis* | 2 | NA | NA | 0,01 | 0,08 |
| *Bacteroides xylanisolvens* | 1 | NA | NA | 0,06 | 0,06 |
| *Barnesiella (genus only)* | 2 | NA | NA | 0,09 | 0,27 |
| *Below cutoff* | 21 | 0,6 | 0,09 | 0,01 | 6,39 |
| *Bifidobacteraceae(G-2) oral taxon 407* | 1 | NA | NA | 0,03 | 0,03 |
| *Bifidobacterium adolescentis* | 1 | NA | NA | 0,01 | 0,01 |
| *Bifidobacterium dentium* | 13 | 0,13 | 0,03 | 0,01 | 2,96 |
| *Bifidobacterium longum* | 13 | 0,42 | 0,3 | 0,01 | 5,01 |
| *Bifidobacterium (genus only)* | 1 | NA | NA | 0,02 | 0,02 |
| *Blautia faecis* | 2 | NA | NA | 0,02 | 0,17 |
| *Blautia (genus only)* | 1 | NA | NA | 0,01 | 0,01 |
| *Blautia wexlerae* | 1 | NA | NA | 0,68 | 0,68 |
| *Brevundimonas vesicularis* | 1 | NA | NA | 0,02 | 0,02 |
| *Bulleidia extructa* | 5 | 0 | 0,02 | 0,01 | 0,06 |
| *Butyrivibrio oral taxon 455* | 4 | 0 | 0,02 | 0,01 | 0,06 |
| *Campylobacter concisus (C. mucosalis)* | 9 | 0,06 | 0,06 | 0,01 | 0,6 |
| *Campylobacter oral taxon 044* | 1 | NA | NA | 0,02 | 0,02 |
| *Campyobacter rectus/showae* | 3 | 0 | 0,03 | 0,02 | 0,04 |
| *Capnocytophaga gingivalis (C. granulosa)* | 2 | NA | NA | 0,01 | 0,15 |
| *Campylobacter gracilis* | 2 | NA | NA | 0,03 | 0,17 |
| *Capnocytophaga (genus only)* | 1 | NA | NA | 0,05 | 0,05 |
| *Capnocytophaga granulosa (C. gingivalis)* | 2 | NA | NA | 0,01 | 0,22 |
| *Capnocytophaga leadbetteri* | 2 | NA | NA | 0,03 | 0,15 |
| *Capnocytophaga oral taxon 336* | 1 | NA | NA | 0,19 | 0,19 |
| *Capnocytophaga oral taxon 338* | 1 | NA | NA | 0,01 | 0,01 |
| *Capnocytophaga sputigena* | 1 | NA | NA | 0,01 | 0,01 |
| *Cardiobacterium hominis* | 2 | NA | NA | 0 | 0,06 |
| *Cardiobacterium valvarum* | 1 | NA | NA | 0,03 | 0,03 |
| *Catonella morbi* | 5 | 0,01 | 0,03 | 0,01 | 0,08 |
| *Clostridium bartlettii* | 4 | 0,03 | 0,14 | 0,01 | 0,4 |
| *Clostridium celatum/disporicum* | 7 | 0,83 | 1,28 | 0,01 | 16,66 |
| *Clostridium glycolicum* | 1 | NA | NA | 0,05 | 0,05 |
| *Clostridium (genus only)* | 3 | 0,26 | 0,27 | 0,15 | 6,47 |
| *Clostridium citroniae/boltae/clostridioforme* | 1 | NA | NA | 0,09 | 0,09 |
| *Clostridium paraputrificum* | 2 | NA | NA | 0,04 | 0,12 |
| *Clostridium perfringens* | 1 | NA | NA | 0,01 | 0,01 |
| *Collinsella aerofaciens* | 1 | NA | NA | 0,06 | 0,06 |
| *Corynebacterium accolens (C. fastidiosum/mcginley/segmentosum/tuberculostearicum)* | 1 | NA | NA | 1,61 | 1,61 |
| *Corynebacterium durum* | 8 | 0,03 | 0,03 | 0,01 | 0,29 |
| *Corynebacterium fastidiosum* | 1 | NA | NA | 0,01 | 0,01 |
| *Corynebacterium (genus only)* | 1 | NA | NA | 0,04 | 0,04 |
| *Corynebacterium matruchotii* | 1 | NA | NA | 0,02 | 0,02 |
| *Corynebacterium proinquum/pseudodiphteriticum* | 2 | NA | NA | 0,01 | 0,42 |
| *Corynebacterium vitaeruminis (pseudotuberculosis/ulcerans/argentoratense)* | 1 | NA | NA | 0,04 | 0,04 |
| *Cryptobacterium curtum* | 5 | 0,01 | 0,03 | 0,02 | 0,06 |
| *Dialister invisus* | 3 | 0 | 0,02 | 0,01 | 0,07 |
| *Dialister micraerophilus* | 1 | NA | NA | 0,01 | 0,01 |
| *Dialister oral taxon 119* | 1 | NA | NA | 0,01 | 0,01 |
| *Dialister pneumosintes* | 1 | NA | NA | 0,01 | 0,01 |
| *Dolosigranulum pigrum* | 2 | NA | NA | 0,03 | 0,04 |
| *Dorea formicigenerans* | 1 | NA | NA | 0 | 0 |
| *Eikenella sp. (NML130454) (Kingella denitrificans)* | 1 | NA | NA | 0,14 | 0,14 |
| *Erysipelotrichaceae(G-1) sp. oral taxon 905* | 1 | NA | NA | 0,06 | 0,06 |
| *Erysipelotrichaceae(G-1) sp. oral taxon 904* | 9 | 0,04 | 0,06 | 0,01 | 0,29 |
| *Erysipelotrichaceae (genus only)* | 5 | 0,02 | 0,02 | 0,01 | 0,41 |
| *Escherichia coli/Shigella boydii/S. dysenteriae/ S flexneri* | 8 | 2,72 | 0,01 | 0,01 | 73,24 |
| *Escherichia/Shigella (genus only)* | 1 | NA | NA | 4,05 | 4,05 |
| *Eubacterium brachy* | 13 | 0,18 | 0,23 | 0,04 | 1,35 |
| *Eubacterium hallii* | 1 | NA | NA | 0,05 | 0,05 |
| *Eubacterium infirmum* | 7 | 0,01 | 0,03 | 0,02 | 0,1 |
| *Eubacterium nodulatum* | 1 | NA | NA | 0,02 | 0,02 |
| *Eubacterium ramulus* | 1 | NA | NA | 0,04 | 0,04 |
| *Eubacterium rectale* | 1 | NA | NA | 0,16 | 0,16 |
| *Eubacterium saphenum* | 2 | NA | NA | 0,01 | 0,15 |
| *Eubacterium sulci (infirmum)* | 12 | 0,12 | 0,29 | 0,03 | 0,65 |
| *Faecalibacterium prausnitzii* | 2 | NA | NA | 0,08 | 0,09 |
| *Filifactor alocis* | 3 | 0 | 0,03 | 0,01 | 0,08 |
| *Flavobacteriaceae genomosp. C1* | 2 | NA | NA | 0,01 | 0,03 |
| *Fretibacterium fastidiosum* | 1 | NA | NA | 0,17 | 0,17 |
| *Fusobacterium necrophorum* | 1 | NA | NA | 0,03 | 0,03 |
| *Fusbacterium nucleatum* | 18 | 0,16 | 0,08 | 0,01 | 1,88 |
| *Fusbacterium periodonticum* | 14 | 1,03 | 0,32 | 0,01 | 17,21 |
| *Fusbacterium (genus only)* | 2 | 0,01 | 0,15 | 0,06 | 0,25 |
| *Gardnerella vaginalis* | 1 | NA | NA | 0,3 | 0,3 |
| *Gemella bergeriae* | 5 | 0,01 | 0,03 | 0,01 | 0,08 |
| *Gemella haemolysans/sanguinis* | 24 | 1,11 | 0,83 | 0,03 | 6,11 |
| *Gemella morbillorum* | 20 | 0,38 | 0,07 | 0,01 | 2,23 |
| *Gemella (genus only)* | 5 | 0,03 | 0,02 | 0,01 | 0,63 |
| *Gemella haemolysans/morbillorum/sanguinis* | 9 | 0,11 | 0,32 | 0,01 | 1,02 |
| *Gemmiger formicilis* | 3 | 0,03 | 0,34 | 0,16 | 0,52 |
| *Gemmiger (genus only)* | 1 | NA | NA | 0,01 | 0,01 |
| *Granulicatella adiacens* | 27 | 8,44 | 5,18 | 0,13 | 23,91 |
| *Granulicatella elegans* | 10 | 0,33 | 0,11 | 0,02 | 6,36 |
| *Granulicatella (genus only)* | 9 | 0,03 | 0,03 | 0,01 | 0,48 |
| *Haemophilus haemolyticus* | 2 | 0,02 | 0,28 | 0,03 | 0,53 |
| *Haemophilus parainfluenzae* | 11 | 0,15 | 0,03 | 0,01 | 1,66 |
| *Haemophilus parahaemolyticus (paraphrohaemolyticus)* | 1 | NA | NA | 0,37 | 0,37 |
| *Haemophilus sp. oral taxon 035* | 1 | NA | NA | 0 | 0 |
| *Haemophilus (genus only)* | 1 | NA | NA | 0,02 | 0,02 |
| *Haemophilus sputorum* | 1 | NA | NA | 0,1 | 0,1 |
| *Helicobacter pylori* | 3 | 0,17 | 0,28 | 0,25 | 3,93 |
| *Holdemania (genus only)* | 1 | NA | NA | 0,02 | 0,02 |
| *Kingella oralis* | 1 | NA | NA | 0,03 | 0,03 |
| *Lachnoanaerobaculum (genus only)* | 2 | NA | NA | 0,02 | 0,02 |
| *Lachnoanaerobaculum oral taxon 089* | 1 | NA | NA | 0,01 | 0,01 |
| *Lachnoanaerobaculum orale/saburreum* | 22 | 0,17 | 0,08 | 0,01 | 1,09 |
| *Lachnoanaerobaculum umaense* | 9 | 0,1 | 0,1 | 0,03 | 1,45 |
| *Lachnospiraceae(G-2) oral taxon 096* | 4 | 0 | 0,03 | 0,02 | 0,03 |
| *Lachnospiraceae (G-3 oral taxon 097)* | 1 | NA | NA | 0,01 | 0,01 |
| *Lachnospiraceae(G-3) oral taxon 100* | 3 | 0,02 | 0,21 | 0,02 | 0,36 |
| *Lachnospiraceae(G-7) oral taxon 163* | 4 | 0,01 | 0,08 | 0,01 | 0,12 |
| *Lactobacillus casei/paracasei/rhamnosus* | 2 | NA | NA | 0,01 | 0,43 |
| *Lactobacillus crispatus/gallinarum (S. acidophilus/ultunensis)* | 1 | NA | NA | 0,33 | 0,33 |
| *Lactobacillus (genus only)* | 4 | 0 | 0,03 | 0,01 | 0,07 |
| *Lactobacillus gasseri* | 6 | 0,58 | 0,51 | 0,04 | 13,38 |
| *Lactobacilus fermentum* | 4 | 0,04 | 0,24 | 0,06 | 0,43 |
| *Lactobacillus reuteri* | 2 | NA | NA | 0,02 | 0,05 |
| *Lactobacillus salivarius* | 2 | NA | NA | 0,01 | 0,36 |
| *Lautrophia mirabilis* | 3 | 0,01 | 0,08 | 0,04 | 0,09 |
| *Leptotrichia (genus only)* | 5 | 0,05 | 0,07 | 0,03 | 0,77 |
| *Leptotrichia goodfellowii* | 1 | NA | NA | 0,01 | 0,01 |
| *Leptotrichia hofstadii* | 1 | NA | NA | 0,04 | 0,04 |
| *Leptotrichia oral taxon 215* | 2 | NA | NA | 0,02 | 0,06 |
| *Leptotrichia oral taxon 218* | 1 | NA | NA | 0,11 | 0,11 |
| *Leptotrichia oral taxon 221* | 2 | NA | NA | 0,04 | 0,06 |
| *Leptotrichia oral taxon 225 (L. buccalis)* | 1 | NA | NA | 0,01 | 0,01 |
| *Leptotrichia sp. oral taxon 417* | 5 | 0,09 | 0,32 | 0,02 | 1,73 |
| *Leptotrichia sp. oral taxon 462* | 3 | 0,04 | 0,08 | 0,02 | 0,89 |
| *Leptotrichia oral taxon 498* | 3 | 0 | 0,01 | 0,01 | 0,02 |
| *Leptotrichia hongkongensis* | 2 | NA | NA | 0,01 | 1,09 |
| *Leptotrichia wadei* | 2 | NA | NA | 0,03 | 0,1 |
| *Megasphaera micronuciformis* | 2 | NA | NA | 0,02 | 0,13 |
| *Mesorhizobium (genus only)* | 1 | NA | NA | 0,22 | 0,22 |
| *Microbacterium (genus only)* | 1 | NA | NA | 0,22 | 0,22 |
| *Micrococcus luteus* | 2 | NA | NA | 0,03 | 0,62 |
| *Mobiluncus (genus only)* | 2 | NA | NA | 0,02 | 0,13 |
| *Mycoplasma salivarium* | 1 | NA | NA | 0,01 | 0,01 |
| *Neisseria bacilliformis* | 1 | NA | NA | 0,02 | 0,02 |
| *Neisseria elongata* | 3 | 0,01 | 0,02 | 0,01 | 0,17 |
| *Neisseria perflava/subflava (cinerea/flavenscens)* | 6 | 0,05 | 0,04 | 0,01 | 0,94 |
| *Neisseria (genus only)* | 1 | NA | NA | 0,09 | 0,09 |
| *Neisseria mucosa/sicca (pharyngis)* | 3 | 0,01 | 0,1 | 0,01 | 0,1 |
| *Odoribacterium splanchnicus* | 1 | NA | NA | 0,05 | 0,05 |
| *Olsenella sp. oral taxon 807* | 1 | NA | NA | 0,02 | 0,02 |
| *Oribacterium asaccharolyticum* | 21 | 0,76 | 0,56 | 0,01 | 7,5 |
| *Oribacterium oral taxon 078* | 6 | 0,01 | 0,02 | 0,01 | 0,03 |
| *Oribacterium (genus only)* | 12 | 0,12 | 0,04 | 0,01 | 2,03 |
| *Oribacterium parvum (sinus)* | 1 | NA | NA | 0,78 | 0,78 |
| *Oribacterium sinus (parvum)* | 21 | 1,16 | 0,63 | 0,03 | 7,33 |
| *Oribacterium (genus only)* | 2 | NA | NA | 0,02 | 0,08 |
| *Oscillibacter sp. (Marseille-P2778)* | 1 | NA | NA | 0,28 | 0,28 |
| *Parabacteroides (genus only)* | 1 | NA | NA | 0,03 | 0,03 |
| *Parascardovia denticolens* | 4 | 0 | 0,03 | 0,02 | 0,06 |
| *Parasutterella excrementihominis* | 2 | NA | NA | 0,1 | 0,71 |
| *Parvimonas micra* | 20 | 0,25 | 0,19 | 0,01 | 1,26 |
| *Parvimonas (genus only)* | 1 | NA | NA | 0,07 | 0,07 |
| *Peptococcus oral taxon 167* | 1 | NA | NA | 0,01 | 0,01 |
| *Peptoniphilus lacrimalis* | 1 | NA | NA | 0,02 | 0,02 |
| *Peptostreptococcaceae(XI)(G-4) oral taxon 369* | 3 | 0 | 0,02 | 0,02 | 0,02 |
| *Peptostreptococcus stomatis* | 10 | 0,02 | 0,03 | 0,01 | 0,1 |
| *Peptostreptococcus (genus only)* | 1 | NA | NA | 0,02 | 0,02 |
| *Porphyromonas endodontalis* | 2 | NA | NA | 0,06 | 0,64 |
| *Porphyromonas (genus only)* | 1 | NA | NA | 0,65 | 0,65 |
| *Porphyromonas oral taxon 279* | 1 | NA | NA | 0,16 | 0,16 |
| *Prevotella denticola* | 1 | NA | NA | 0,02 | 0,02 |
| *Prevotella (genus only)* | 1 | NA | NA | 0,09 | 0,09 |
| *Prevotella histicola* | 4 | 0,03 | 0,08 | 0,02 | 0,52 |
| *Prevotella melaninogenica* | 4 | 0,08 | 0,56 | 0,03 | 1,14 |
| *Prevotella nanceiensis* | 1 | NA | NA | 0,1 | 0,1 |
| *Prevotella nigrescens* | 1 | NA | NA | 0,03 | 0,03 |
| *Prevotella oral taxon 304* | 1 | NA | NA | 0,06 | 0,06 |
| *Prevotella oral taxon 306* | 2 | NA | NA | 0,02 | 0,14 |
| *Prevotella oral taxon 317 (P. conceptionensis)* | 2 | NA | NA | 0,01 | 0,02 |
| *Prevotella oris* | 1 | NA | NA | 0,03 | 0,03 |
| *Prevotella pallens* | 1 | NA | NA | 0,47 | 0,47 |
| *Prevotella pleuritidis* | 1 | NA | NA | 0,02 | 0,02 |
| *Prevotella salivae* | 2 | NA | NA | 0,17 | 0,18 |
| *Propionibacterium acidifaciens* | 1 | NA | NA | 0,04 | 0,04 |
| *Pseudoflavonifractor (genus only)* | 1 | NA | NA | 0,01 | 0,01 |
| *Pseudomonas (genus only)* | 4 | 0,01 | 0,02 | 0,01 | 0,11 |
| *Pseudomonas putida* | 1 | NA | NA | 0,01 | 0,01 |
| *Pseudomonas stutzeri* | 1 | NA | NA | 0,65 | 0,65 |
| *Reyranella (genus only)* | 3 | 0,01 | 0,02 | 0,02 | 0,26 |
| *Reyranella soli* | 1 | NA | NA | 0,03 | 0,03 |
| *Romboutsia timonensis* | 6 | 1,53 | 4,91 | 0,26 | 18 |
| *Romboutsia (genus only)* | 3 | 0,11 | 0,85 | 0,3 | 1,75 |
| *Roseburia intestinalis (R. hominis)* | 1 | NA | NA | 0,06 | 0,06 |
| *Rosebruria hominis (R. intestinalis)* | 1 | NA | NA | 0,02 | 0,02 |
| *Roseburia faecis* | 1 | NA | NA | 0,02 | 0,02 |
| *Roseburia inulinivorans* | 3 | 0 | 0,02 | 0,01 | 0,03 |
| *Rothia aeria* | 5 | 0,04 | 0,05 | 0,02 | 0,71 |
| *Rothia dentocariosa* | 20 | 0,74 | 0,36 | 0,04 | 4,71 |
| *Rothia (genus only)* | 11 | 0,05 | 0,04 | 0,02 | 0,76 |
| *Rothia mucilaginosa* | 26 | 9,07 | 6,55 | 0,33 | 48,96 |
| *Ruminococcaceae(G-1) (genus only)* | 1 | NA | NA | 0,09 | 0,09 |
| *Ruminococcaceae(G-1) sp. oral taxon 075* | 6 | 0,55 | 0,1 | 0 | 14,03 |
| *Ruminococcaceae(G-2) sp. oral taxon 085* | 11 | 0,11 | 0,09 | 0,01 | 1,67 |
| *Ruminococcus faecis* | 1 | NA | NA | 0,18 | 0,18 |
| *Ruminococcus gnavus* | 2 | NA | NA | 0,06 | 1,68 |
| *Ruminococcus lactaris* | 1 | NA | NA | 0,51 | 0,51 |
| *Ruminococcus bromii* | 2 | NA | NA | 0,03 | 0,2 |
| *Ruminococcus torques* | 1 | NA | NA | 0,28 | 0,28 |
| *Ruminococus (genus only)* | 1 | NA | NA | 0,02 | 0,02 |
| *Scardovia (genus only)* | 1 | NA | NA | 0,59 | 0,59 |
| *Scardovia inopinata* | 1 | NA | NA | 0,01 | 0,01 |
| *Scardovia wiggsiae* | 12 | 0,23 | 0,2 | 0,01 | 1,73 |
| *Shuttleworthia satelles* | 5 | 0,01 | 0,02 | 0,01 | 0,06 |
| *Slackia exugia* | 1 | NA | NA | 0,01 | 0,01 |
| *Solobacterium moorei* | 26 | 0,64 | 0,45 | 0,04 | 3,51 |
| *Solobacterium (genus only)* | 6 | 0 | 0,02 | 0,01 | 0,02 |
| *Sp.hingobium (genus only)* | 1 | NA | NA | 0,04 | 0,04 |
| *Staphylococcus (genus only)* | 1 | NA | NA | 0,03 | 0,03 |
| *Stenotrophomonas maltophila* | 2 | NA | NA | 0,05 | 1,96 |
| *Stomatobaculum sp. oral taxon 097* | 8 | 0,01 | 0,02 | 0,01 | 0,1 |
| *Stomatobaculum sp. oral taxon 373* | 1 | NA | NA | 0,02 | 0,02 |
| *Stomatobaculum longum* | 10 | 0,03 | 0,03 | 0,02 | 0,29 |
| *Streptococcus anginosus group* | 23 | 0,81 | 0,16 | 0,01 | 8,05 |
| *Streptococcus (genus only)* | 26 | 0,87 | 0,5 | 0,01 | 6,12 |
| *Streptococcus mitis group* | 27 | 26,7 | 20,74 | 0,36 | 77,15 |
| *Streptococcus mutans group* | 8 | 0,07 | 0,05 | 0,01 | 1,21 |
| *Streptococcus salivarius group* | 22 | 4,13 | 2,72 | 0,1 | 14,49 |
| *Streptococcus sanguinis group* | 27 | 17,89 | 17,47 | 0,15 | 41,27 |
| *Tannerella oral taxon 286* | 3 | 0 | 0,01 | 0,01 | 0,05 |
| *Tannerella forsythia* | 1 | NA | NA | 0,01 | 0,01 |
| *Tetragenococcus halophilus* | 1 | NA | NA | 0,02 | 0,02 |
| *TM7(G-1) (genus only)* | 21 | 0,38 | 0,24 | 0,02 | 3,91 |
| *TM7(G-1) oral taxon 346* | 17 | 0,29 | 0,05 | 0,01 | 4,6 |
| *TM7(G-1) oral taxon 347* | 6 | 0,04 | 0,09 | 0,01 | 0,72 |
| *TM7(G-1) oral taxon 348* | 2 | NA | NA | 0,04 | 0,12 |
| *TM7(G-1) oral taxon 349* | 2 | NA | NA | 0,01 | 0,02 |
| *TM7(G-1) oral taxon 352/TM7x* | 24 | 2,73 | 0,62 | 0,02 | 23,32 |
| *TM7(G-1) oral taxon 488* | 2 | NA | NA | 0,01 | 0,03 |
| *TM7(G-2) oral taxon 350* | 3 | 0 | 0,02 | 0,01 | 0,02 |
| *TM7 (G-3) oral taxon 351* | 8 | 0,02 | 0,04 | 0,01 | 0,26 |
| *TM7 (G-3) (genus only)* | 1 | NA | NA | 0,01 | 0,01 |
| *TM7(G-4) oral taxon 355* | 2 | NA | NA | 0,02 | 0,07 |
| *TM7(G-5) sp. oral taxon 356* | 7 | 0,01 | 0,01 | 0,01 | 0,16 |
| *TM7(G-6) sp. oral taxon 870* | 8 | 0,25 | 0,69 | 0,26 | 2,14 |
| *TM7(G-6) (genus only)* | 5 | 0,34 | 0,22 | 0,08 | 5,6 |
| *Treponema medium/vincentii* | 1 | NA | NA | 0,01 | 0,01 |
| *Tropheryma whipplei* | 3 | 0,04 | 0,18 | 0,12 | 0,84 |
| *Turicibacter (genus only)* | 2 | NA | NA | 0,01 | 0,02 |
| *Turicibacter sanguinis* | 4 | 0,07 | 0,54 | 0,04 | 0,71 |
| *Veillonella atypica* | 1 | NA | NA | 0,01 | 0,01 |
| *Veillonella dispar (V. parvula)* | 9 | 0,01 | 0,03 | 0 | 0,09 |
| *Veillonella (genus only)* | 8 | 0,01 | 0,02 | 0,01 | 0,03 |
| *Veillonella parvula (V. tobetsuensis/dentocariosa)* | 1 | NA | NA | 0,01 | 0,01 |
|  |  |  |  |  |  |

**Supplementary Table S3: Specific amplification and Sanger sequencing of selected gdh and rpoB -gene targets. Results per sample and per target**

| Sample  ID | *S. mitis*  (gdh) | *S. oralis*  (gdh) | *S. cristatus*  (gdh) | *S. gordonii*  (gdh) | *S. salivarius* and  *S. vestibularis*  (gdh) | *S. parasanguinis*  (gdh) | *G. haemolysans*  (rpoB) | *G. haemolysans group*  (rpoB) | *G. sanguinis*  (rpoB) |
| --- | --- | --- | --- | --- | --- | --- | --- | --- | --- |
| 1 | mitis | oralis | cristatus | gordonii | salivarius | parasanguinis | - | parahaemolysans | sanguinis |
| 2 | mitis | oralis | - | - | salivarius | parasanguinis | - | - | sanguinis |
| 3 | mitis | oralis | cristatus | gordonii | salivarius | parasanguinis | haemolysans | taiwanensis | sanguinis |
| 4 | mitis | oralis |  | gordonii | - | - | - | - | - |
| 5 | mitis | oralis | cristatus | gordonii | salivarius | parasanguinis | - | parahaemolysans | sanguinis |
| 6 | - | oralis | - | - | salivarius | - | - | - | - |
| 8 | - | - | - | - | salivarius | parasanguinis | - | - | - |
| 9 | mitis | oralis | cristatus | - | salivarius | parasanguinis | - | - | sanguinis |
| 10 | mitis | oralis | - | gordonii | salivarius | parasanguinis | haemolysans | taiwanensis | sanguinis |
| 11 | mitis | oralis | cristatus | - | salivarius | parasanguinis | - | taiwanensis | - |
| 12 | mitis | oralis | - | - | - | - | - | parahaemolysans | - |
| 13 | - | oralis | - | gordonii | - | parasanguinis | - | - | - |
| 14 | mitis | oralis | cristatus | gordonii | salivarius | parasanguinis | - | taiwanensis | - |
| 15 | mitis | oralis | cristatus | gordonii | salivarius | parasanguinis | haemolysans | - | sanguinis |
| 16 | mitis | - | - | gordonii | - | parasanguinis | haemolysans | - | - |
| 18 | mitis | oralis | - | gordonii | salivarius | parasanguinis | - | parahaemolysans | sanguinis |
| 19 | mitis | - | - | - | - | parasanguinis | haemolysans | - | - |
| 20 | - | - | cristatus | - | salivarius | parasanguinis | - | - | - |
| 21 | mitis | oralis | cristatus | gordonii | salivarius | parasanguinis | - | parahaemolysans | - |
| 22 | mitis | oralis | cristatus | - | salivarius | parasanguinis | haemolysans | - | sanguinis |
| 23 | mitis | - | - | - | - | - | - | - | - |
| 24 | mitis | oralis | - | gordonii | salivarius | parasanguinis | - | parahaemolysans | sanguinis |
| 25 | mitis | oralis | cristatus | gordonii | salivarius | parasanguinis | - | - | sanguinis |
| 26 | mitis | oralis | cristatus | - | salivarius | parasanguinis | - | taiwanensis | sanguinis |
| 27 | mitis | oralis | cristatus | gordonii | salivarius | parasanguinis | - | parahaemolysans | sanguinis |
| 28 | mitis | oralis | cristatus | gordonii | salivarius | parasanguinis | - | parahaemolysans | - |
| 29 | mitis | oralis | cristatus | gordonii | salivarius | parasanguinis | - | parahaemolysans | sanguinis |
| 30 | mitis | - | - | - | salivarius | parasanguinis | haemolysans | taiwanensis | - |
| Total  positive | 24 | 22 | 15 | 16 | 22 | 24 | 7 | 15 | 14 |
| Homology (%) | 97.5-100 | 94.3-100 | 94.1-100 | 97.9-100 | 100 | 97.5-100 | 98.2-99.1 | 98.5-100 | 99.1-100 |

Dash-sign (-): target not detected.

Homology (%): Range of observed % homologies with closest reference for all samples. Due to lack of established cutoff-values for a valid species-level assignment, all identifications were supported by a pairwise comparison of the alignment table in GenBank using the “distance tree of results” function.

**Supplementary table S4. Alpha diversities**

| SAMPLE | Shannon index  Average 2,84  Mean 20,86 | Species richness  Average 51  Mean 50 |
| --- | --- | --- |
| 1 | 2,70 | 46 |
| 2 | 2,75 | 42 |
| 3 | 2,78 | 54 |
| 4 | 1,76 | 15 |
| 5 | 3,26 | 48 |
| 6 | 3,34 | 30 |
| 8 | 2,36 | 66 |
| 9 | 3,33 | 54 |
| 10 | 2,89 | 51 |
| 11 | 2,85 | 58 |
| 12 | 0,83 | 16 |
| 13 | 3,34 | 46 |
| 14 | 3,07 | 79 |
| 15 | 2,71 | 124 |
| 16 | 2,79 | 33 |
| 18 | 2,86 | 57 |
| 19 | 2,50 | 34 |
| 20 | 3,20 | 58 |
| 21 | 2,86 | 41 |
| 22 | 3,09 | 57 |
| 24 | 3,36 | 50 |
| 25 | 2,77 | 41 |
| 26 | 2,89 | 44 |
| 27 | 3,72 | 66 |
| 28 | 1,48 | 44 |
| 29 | 3,62 | 52 |
| 30 | 3,62 | 81 |

**Supplementary Table S5: A comparison between RipSeq NGS (5b) and QIIME (5c) for the analysis of a commercial bacterial mock community (5a)**

Summary: RipSeq NGS identifies all the mock community species to the best possible level within the limitations defined by the resolution of the 16S rRNA gene itself. QIIME identifies most of the sequences to the genus, family or order-level only. It also provides several erroneous or inaccurate species level designations. Both pipelines find fewer *Rhodobacter sphaeroides* sequences than expected, most likely reflecting inefficient amplification or sequencing of this target. The RipSeq NGS pipeline underestimates the number of *Staphylococcus aureus* sequences when OUT-clustering is performed with a 99 % similarity threshold. This is because *Staphylococcus aureus* shares 99.3 % homology with *Staphylococcus epidermidis* leading to most of the reads being erroneously assigned to the *S. epidermidis* cluster. This is a well-known issue when using de-novo OTU clustering for very similar species. As demonstrated in table S6b the problem can be diminished by using a higher similarity threshold of 99.5 %. For the study we nevertheless used a OTU-clustering of 99 % since using a higher threshold also increased the number of small groups representing out-layer sequences and consequently increased the workload in the quality assuring of the data-analysis.

**Table S5a**: The content of the commercial mock community

| **Mock community species** | **operons** | **% dist.** |
| --- | --- | --- |
| *Rhodobacter sphaeroides* | 1000000 | 21.91 % |
| *Staphylococcus epidermidis* | 1000000 | 21.91 % |
| *Streptococcus mutans* | 1000000 | 21.91 % |
| *Escherichia coli* | 1000000 | 21.91 % |
| *Bacillus cereus* | 100000 | 2.19 % |
| *Clostridium beijerinckii* | 100000 | 2.19 % |
| *Pseudomonas aeruginosa* | 100000 | 2.19 % |
| *Staphylococcus aureus* | 100000 | 2.19 % |
| *Streptococcus agalactiae* | 100000 | 2.19 % |
| *Acinetobacter baumannii* | 10000 | 0.22 % |
| *Helicobacter pylori* | 10000 | 0.22 % |
| *Lactobacillus gasseri* | 10000 | 0.22 % |
| *Listeria monocytogenes* | 10000 | 0.22 % |
| *Neisseria meningitidis* | 10000 | 0.22 % |
| *Propionibacterium acnes* | 10000 | 0.22 % |
| *Acitinomyces odontolyticus* | 1000 | 0.02 % |
| *Bacteroides vulgatus* | 1000 | 0.02 % |
| *Deinococcus radiodurans* | 1000 | 0.02 % |
| *Enterococcus faecalis* | 1000 | 0.02 % |
| *Streptococcus pneumoniae* | 1000 | 0.02 % |
|  | 4565000 | 100.00 % |

Mock community: HM-783D BEI Resources (Virginia, US)

% dist = relative distribution of reads in percent

**Table S5b**: Results obtained using RipSeq NGS

| **Mock community species** | **RipSeq NGS result** | **Reads**  **99 %** | **% dist.**  **99 %** | **% dist.**  **99.5 %** |
| --- | --- | --- | --- | --- |
| *Rhodobacter sphaeroides* | *R. johrii /megalophilus /sphaeroides* | 7793 | 6.01%(L) | 5.96%(L) |
| *Staphylococcus epidermidis* | *S. capitis/caprae/epidermidis (aureus/cohnii/hominis/lugdunensis/*  *saccharolyticus)* | 38075 | 29.38% | 27.52% |
| *Streptococcus mutans* | *S. mutans* | 33725 | 26.02% | 25.97% |
| *Escherichia coli* | *E. coli/Shigella spp.* | 35979 | 27.76% | 27.85% |
| *Bacillus cereus* | *B. anthracis/cereus/thuringiensis* | 4003 | 3.09% | 3.06% |
| *Clostridium beijerinckii* | *C. beijerinckii (puniceum)* | 2703 | 2.09% | 2.11% |
| *Pseudomonas aeruginosa* | *P. aeruginosa* | 1469 | 1.13% | 1.14% |
| *Staphylococcus aureus* | *S. aureus (croceolyticum/petrasii)* | 102 | 0.08%(L) | 2.01% |
| *Streptococcus agalactiae* | *S. agalactiae* | 4119 | 3.18% | 3.11% |
| *Acinetobacter baumannii* | *A. baumannii* | 305 | 0.24% | 0.24% |
| *Helicobacter pylori* | *H. pylori* | 358 | 0.28% | 0.27% |
| *Lactobacillus gasseri* | *L. gasseri (johnsonii)* | 216 | 0.17% | 0.16% |
| *Listeria monocytogenes* | *L. innocua/ivanovii/marthii/ monocytogenes /seeligeri/welshimeri* | 333 | 0.26% | 0.26% |
| *Neisseria meningitidis* | *N. meningitidis (polysaccharea)* | 221 | 0.17% | 0.17% |
| *Propionibacterium acnes* | *P. acnes* | 145 | 0.11% | 0.11% |
| *Acitinomyces odontolyticus* | *A. meyeri/odontolyticus group* | 9 | 0.01% | 0.01% |
| *Bacteroides vulgatus* | *B. vulgatus* | 11 | 0.01% | 0.01% |
| *Deinococcus radiodurans* | *D. radiodurans* | 20 | 0.02% | 0.02% |
| *Enterococcus faecalis* | *E. faecalis* | 19 | 0.01% | 0.01% |
| *Streptococcus pneumoniae* | *S. pneumoniae/pseudopneumoniae (mitis/oralis/infantis)* | 8 | 0.01% | 0.01% |
|  | Total | 129613 | 100.00% | 100.00% |

Results obtained using the RipSeq NGS software and a cluster similarity of 99 %. The % distribution of reads is also showed for an analysis using a cluster similarity of 99.5 %.

% dist = relative distribution of reads in percent

Slash-results: Species with identical 16S rRNA-genes in the sequenced area. No discrimination possible.

Results in parenthesis: species within 0.8 % from the top-scoring sequence. Robust discrimination not possible.

L = significantly lower than expected

**Table S5c**: Results obtained using QIIME

| **Mock community species** | **QIIME-result** | **reads** | **% dist.** |
| --- | --- | --- | --- |
| *Rhodobacter sphaeroides* | *R. sphaeroides^* | 9780 | 6.29%(L) |
| *Staphylococcus epidermidis* | *S. epidermidis^* | 302 | 26.30% |
|  | *Staphylococcus* (g) | 40608 |  |
| *Streptococcus mutans* | *Streptococcus* (g)* | 40596* | 26.10%* |
| *Escherichia coli* | *E. coli^* | 18 | 27.69% |
|  | *Enterobacteriaceae* (f) | 43051 |  |
| *Bacillus cereus* | *B. cereus^§^* | 163 | 4.07% |
|  | *B. alkalitolerans* (E) | 20 |  |
|  | *B. anthracis*^§^ | 10 |  |
|  | *Bacillus* (g) | 4847 |  |
|  | *Bacillales* (f) | 1295 |  |
| *Clostridium beijerinckii* | *C. butyricum* (E) | 40 | 2.28% |
|  | *Clostridiales* (o) | 3510 |  |
| *Pseudomonas aeruginosa* | *P. alcaligenes* (E) | 1712 | 1.44% |
|  | *Pseudomonas* (g) | 532 |  |
| *Staphylococcus aureus* | *S. aureus* | 5030 | 3.23% |
| *Streptococcus agalactiae* | *Streptococcus* (g) | * | * |
| *Acinetobacter baumannii* | *Acinetobacter* (g) | 486 | 0.31% |
| *Helicobacter pylori* | *H. pylori* | 510 | 0.36% |
|  | *H. pullorum* (E) | 41 |  |
| *Lactobacillus gasseri* | *Lactobacillus* (g) | 318 | 0.20% |
| *Listeria monocytogenes* | *L. seelingeri*^#^ | 447 | 0.29% |
| *Neisseria meningitidis* | *N. cincerae* (E) | 324 | 0.21% |
| *Propionibacterium acnes* | *P. acnes* | 211 | 0.14% |
| *Acitinomyces odontolyticus* | *Actinomyces* (g) | 13 | 0.01% |
| *Bacteroides vulgatus* | *Bacteroides* (g) | 27 | 0.02% |
| *Deinococcus radiodurans* | *Deinococcus* (g) | 25 | 0.02% |
| *Enterococcus faecalis* | *Enterococcus* (g) | 42 | 0.03% |
| *Streptococcus pneumoniae* | *Streptococcus* (g) | * | * |
|  | *Gemellales* (o) (E) | 184 | 0.12% |
|  | *Planococcaceae* (f) (E) | 1418 | 0.91% |
| Total |  | 155560 | 100.00% |

Results obtained using the QIIME bioinformatics pipeline version 1.9.1 with command “pick_closed_reference_otus.py” and default 97 % cutoff for species identification.

% dist = relative distribution of reads in percent

E = Erroneous identification or species assignment, L = significantly lower than expected f = family-level, g = genus-level, o = order-level

*No discrimination between S. agalactiae, S. mutans and S. pneumoniae. All merged into single genus-level identification

^Unambiguous identification is not possible, since this species is identical to other species in the sequenced area. It should have been a slash-result. Please refer to table S6b.

^§^B. anthracis and B. cereus are identical in the sequenced area. Should have been a slash-result B. cereus/anthracis

^#^L. seelingeri is identical to L. monocytogenes in the sequenced area. Should have been a slash-result L. seelingeri/monocytogenes.

**Supplementary Table S6. Primer sequences and PCR conditions for targeted Sanger-sequencing of the rpoB and gdh genes**

| **Target species** | **Gene** | **Primers** | **Conc.**  **(µM)** | **Anneal.**  **(****ºC)** |
| --- | --- | --- | --- | --- |
| *G. haemolysans* | rpoB | Forward: 5’-AGGAATCATTCGTATTGG-3’  Reverse: 5’-AACATCTTCATCAGTAGC-3’ | 0.4  0.4 | 56 |
| *G. haemolysans*  *G. parahaemolysans*  *G. taiwanensis* | rpoB | Forward: 5’-TAAAGTTACACCGAAAGG-3’  Reverse: 5’-CATCAAATACTGGTGTTG-3’ | 0.4  0.4 | 56 |
| *G. sanguinis* | rpoB | Forward: 5’-GTGGTATTATTCGTATAGGT-3’  Reverse: 5’-CATCAAATACTGGTGTTG-3’ | 0.4  0.4 | 56 |
| *S. cristatus* | gdh | Forward: 5’-CTAATCTGCTGTTTGAAA-3’  Reverse: 5’-GATATAGATGGTTAGGACA-3’ | 0.4  0.4 | 53 |
| *S. gordonii* | gdh | Forward: 5’-AGCYAATAATTCTGCTGAAG-3’  Reverse: 5’-CAGAATCTATGACTGAAACTT-3’ | 0.4  0.4 | 57 |
| *S. mitis* | gdh | Forward: 5’-ATGAAGARCTTAAAGAAYACT-3’  Reverse: 5’-TGGCTAAAGTTAGTTGAGT-3’ | 0.6  0.4 | 53 |
| *S. oralis* | gdh | Forward: 5’-CTGAGGAAGAAYTGAAAGAA-3’  Reverse: 5’-ATTGGTWGARTTGTTRTTCA-3’ | 0.4  0.6 | 53 |
| *S. parasanguinis* | gdh | Forward: 5’-CTTTACYAAGGATGCRATTC-3’  Reverse: 5’-GATCCAGACTTGTATTCATAGA-3’ | 0.6  0.4 | 55 |
| *S. salivarius*  *S. vestibularis* | gdh | Forward: 5’-TTGGGAGCTATTGATGTC-3’  Reverse: 5’-GCRTCTGCAACTTTAAGG-3’ | 0.4  0.4 | 57 |

Conc.: Final concentration in the 25 µl PCR-reaction tube, Anneal.: PCR-specific annealing temperature
